# A retrospective comparison of CD19 single and CD19/CD22 bispecific targeted chimeric antigen receptor T cell therapy in patients with relapsed/refractory acute lymphoblastic leukemia

**DOI:** 10.1038/s41408-020-00371-6

**Published:** 2020-10-19

**Authors:** Yiyun Wang, Yingying Yang, Ruimin Hong, Houli Zhao, Guoqing Wei, Wenjun Wu, Huijun Xu, Jiazhen Cui, Yanlei Zhang, Alex H. Chang, Yongxian Hu, He Huang

**Affiliations:** 1 Bone Marrow Transplantation Center, The First Affiliated Hospital, Zhejiang University School of Medicine, Zhejiang, China; 2 Institute of Hematology, Zhejiang University, Zhejiang, China; 3 Zhejiang Province Engineering Laboratory for Stem Cell and Immunity Therapy, Zhejiang, China; 4 Zhejiang Laboratory for Systems & Precision Medicine, Zhejiang University Medical Center, Zhejiang, China; 5 Shanghai YaKe Biotechnology Ltd, Shanghai, China

**Keywords:** Immunotherapy, Cancer immunotherapy

Dear Editor,

CD19 chimeric antigen receptor T cell (CAR-T) therapy has achieved high response rates in patients with relapsed/refractory acute lymphoblastic leukemia (R/R ALL). However, it was reported that approximately 50% of patients who achieved complete remission (CR) eventually relapsed in 1 year^1^. Therefore, rational prophylaxis against the escape of CD19-negative tumors is to generate T cells capable of recognizing multiple antigens^2^. In addition to CD19, CD22 is another member of the B cell antigen family that has been validated as a successful target for B cell leukemias^3,4^. Hence, we presented a CD19 and CD22 bispecific CAR, which is under investigation in clinical studies.

CARs were constructed as described in previous reports^5,6^ (Supplementary Fig. 1). Notably, similar to its CAR-T signaling domain counterpart^7^, CAR structure has a significant impact on mediating transduction, cytokine production, and cytotoxicity, which correspondingly impacts clinical outcomes. Hence, this article retrospectively analyzed in vitro characteristics and in vivo kinetic differences between CD19 and CD19/CD22 bispecific CAR-T.

All patients enrolled in the CD19 CAR-T clinical trials (ChiCTR-ORN-16008948, *n* = 35) and CD19/22 CAR-T clinical trials (ChiCTR1800015575, *n* = 15) from 1 July 2015 to 30 April 2020 were selected for this retrospective series. None of the patients had previously been treated with any cell immunotherapy or Bite CD19 and CD22 antibody-drug conjugates. Other baseline characteristics are summarized in Supplementary Table 1.

Before 1 October 2017, patients had not continuously observed the subtype of their cells by flow cytometry in the early stage. Hence, eight patients received CD19 CAR-T therapy, and 15 patients received CD19/CD22 CAR-T therapy later and were analyzed for the transduction and differentiation characteristics of the two types of CAR-T cells (Supplementary Table 2). The average transduction rate of CD19 CAR-T cells was higher than that of the CD19/CD22 CAR-T cells (45.5% vs 35.9%, *P* = 0.038), which shows that the CAR sequence did affect the initial transduction rate because the CD19/CD22 CAR had a larger size (Supplementary Fig. 2a). Moreover, during the cultivation process, there was no difference in the cell growth rate between the two groups. Furthermore, we analyzed the cell differentiation phenotype of CAR-T cells between the two groups. The median ratio of CD4+ to CD8+ CAR-T cells was 0.92 (range, 0.57–2.70) and 0.52 (range, 0.07–3.45), respectively. A slight but statistically significant increase in the CD4+ naïve T cell (Tn) population was consistently observed in cultured CD19/CD22 CAR-T cells compared with CD19 CAR-T cells (30.6% ± 29.0 vs 57.5% ± 20.9, *P* = 0.024), and fewer CD8+ effector T cells were observed in the CD19/CD22 CAR-T group (0.24% vs 0.03%, *P* = 0.029, Supplementary Fig. 2b).

The peripheral blood samples of patients were continuously tested using flow cytometry. On average, the CD19 CAR-T group expanded >1% on day 4 (range, 1–6) and the CD19/CD22 CAR-T group expanded on day 3 (range, 2–10) after infusion. They achieved the peak number on day 10.5 (CD19 CAR-T group, within a range of 8–13 days) and day 9 (CD19/CD22 CAR-T group, within a range of 5–14 days), which lasted 33.5 days (CD19 CAR-T group, within a range of 12–74 days) and 24.5 days (CD19/CD22 CAR-T group, within a range of 15–77 days), respectively (Supplementary Figs. 3a–c). The median peak CAR-T number was 590.4 cells/μL (range, 76.0–2102.1 cells/μL) and 448.2 cells/μL (range, 63.5–4142.6 cells/μL) in the CD19 CAR-T group and CD19/CD22 CAR-T group, respectively (Supplementary Fig. 3d).

The baseline characteristics of 35 patients who received CD19 CAR-T and 15 patients who received CD19/CD22 CAR-T are summarized in Supplementary Table 3. A sensitivity of 0.01% for Minimal residual disease (MRD) was achieved in all samples analyzed after 1 month of CAR-T infusion. The CR rate was 91.4% (32/35) in patients who received CD19 CAR-T cells and 86.7% (13/15) in patients who received CD19/CD22 CAR-T cells. Further, MRD^−^ CR rates were 88.6% (31/35) and 86.7% (13/15), respectively (*P* = 0.630).

There were significantly more patients who developed severe cytokine release syndrome (CRS, Grade ≥ 3) in the CD19 CAR-T group than in the CD19/CD22 CAR-T group (*P* = 0.029, Fig. 1a). Notably, CD19 CAR-T was found to be an independent risk factor associated with severe CRS (odds ratio: 0.183, 95% confidence interval (CI): 0.036–0.933, *P* = 0.041, Supplementary Table 4). Moreover, 5.7% (2/35) of patients suffered from CAR-T cell-related encephalopathy syndrome (CRES) in the CD19 CAR-T group, while none had CRES in the CD19/CD22 CAR-T group (Supplementary Table 5). In addition, 98% (49/50) of patients enrolled in this study had fever after infusion of CAR-T cells. The median duration of fever was 8 days (range, 2–45 days) in the CD19 CAR-T group and 7 days (range, 3–40 days) in the CD19/CD22 CAR-T group (Fig. 1b). However, there was no statistically significant difference in the peak values of D-dimer, ferritin, interleukin-2 (IL-2), IL-4, IL-6, IL-10, tumor necrosis factor, interferon-γ, and IL-17A between the CD19 and CD19/CD22 CAR-T groups. Moreover, patients in the CD19 CAR-T group had fewer minimum neutrophils and experienced a longer duration of neutropenia (Supplementary Fig. 4). Otherwise, 8 (22.9%) patients and 2 (13.3%) patients received tocilizumab only in the CD19 and CD19/22 CAR-T groups, respectively. Three (8.6%) patients and 2 (13.3%) patients received tocilizumab and glucocorticoid in the CD19 and CD19/22 CAR-T groups, respectively (Supplementary Table 3).

**Fig. 1:**
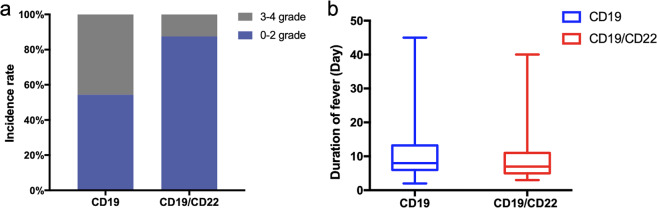
Toxicities in CD19 single and CD19/CD22 dual target CAR-T group. **a** Groups according to the severity of CRS. The stacked bars describe the incidence rate of severe CRS. **b** The box graph shows the duration of fever in the two groups.

Depending on the willingness, economic background, and quality of life of patients, some patients underwent haplo-HSCT after CAR-T infusion. Up to the final follow-up (1 June 2020), among the nontransplant patients in the CD19-targeting CAR-T group, 73.7% (14/19) of patients relapsed, and the median leukemia-free survival (LFS) time was 60 days (range, 30–261 days). Otherwise, among four nontransplant patients in the CD19/CD22 group, three of four patients relapsed at 60, 90, and 110 days after receiving CAR-T treatment (Supplementary Table 6). There was no significant difference in LFS between the CD19 CAR-T group (65 days [95% CI, 0–183 days]) and the CD19/CD22 CAR-T group (90 days [95% CI, 41–139 days], Fig. 2a). Up to the final follow-up, no significant difference was observed in terms of overall survival time (OS) between the CD19 CAR-T group (364 days [95% CI, 254.6–473.4 days]) and the CD19/CD22 CAR-T group (652 days [95% CI, 390.0–905.0 days], *P* = 0.198, Fig. 2b).

**Fig. 2:**
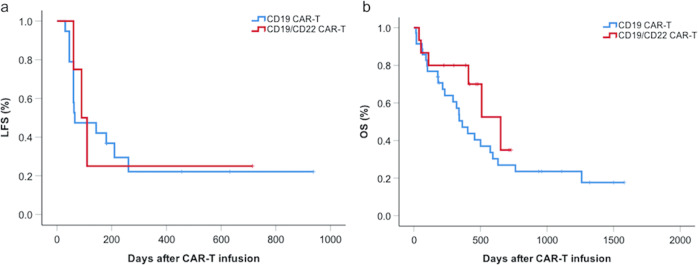
Long-term survival in CD19 single and CD19/CD22 dual target CAR-T group. **a** LFS between the CD19 CAR-T group and the CD19/CD22 CAR-T group. **b** OS between the CD19 CAR-T group and the CD19/CD22 CAR-T group.

In conclusion, we compared the characteristics and clinical outcomes of CD19 single and CD19/CD22 bispecific targeted CAR-T cells in 50 patients with R/R ALL in this retrospective study. We found that CD19/CD22 dual-target CAR-T cells had slight CRS toxicity (*P* = 0.029), which is more suitable for the elderly, severe patients or patients with high tumor burdens. Unfortunately, CD19/CD22 dual-target CAR-T cells demonstrated a comparable CR rate with that of CD19 CAR-T cells and did not reduce the recurrence rate in R/R ALL. For adult patients who achieve CR after CAR-T cell therapy, allo-HSCT may be a reasonable option^8^.

## Supplementary information

A retrospective comparison of CD19 single and CD19/CD22 bispecific targeted chimeric antigen receptor T cell therapy in patients with relapsed/refractory acute lymphoblastic leukemia
