# Supplementary material for: A retrospective comparison of CD19 single and CD19/CD22 bispecific targeted chimeric antigen receptor T cell therapy in patients with relapsed/refractory acute lymphoblastic leukemia
Source: Blood Cancer J. 2020 Oct 19;10(10):105. doi: 10.1038/s41408-020-00371-6 (PMC7572410; doi:10.1038/s41408-020-00371-6)
Supplement: Supplementary file 1 — A retrospective comparison of CD19 single and CD19/CD22 bispecific targeted chimeric antigen receptor T cell therapy in patients with relapsed/refractory acute lymphoblastic leukemia [file 41408_2020_371_MOESM1_ESM.docx]

**Supplementary materials**

**A retrospective comparison of CD19 single and CD19/CD22 bispecific targeted chimeric antigen receptor T cell therapy in patients with relapsed/refractory acute lymphoblastic leukemia**

Yiyun Wang^1,2,3,4*^, Yingying Yang^1,2,3,4*^, Ruimin Hong^1,2,3,4*^, Houli Zhao^1,2,3,4^, Guoqing Wei^1,2,3,4^, Wenjun Wu^1,2,3,4^, Huijun Xu^1,2,3,4^, Jiazhen Cui^1,2,3,4^, Yanlei Zhang^5^, Alex H. Chang^5^, Yongxian Hu^1,2,3,4#^, He Huang^1,2,3,4#^

^1^ Bone Marrow Transplantation Center, the First Affiliated Hospital, Zhejiang University School of Medicine；

^2^ Institute of Hematology, Zhejiang University；

^3^Zhejiang Province Engineering Laboratory for Stem Cell and Immunity Therapy；

^4^Zhejiang Laboratory for Systems & Precision Medicine, Zhejiang University Medical Center；

^5^Shanghai YaKe Biotechnology Ltd.

*** These authors contributed equally to this work.**

**# Corresponding author:** Prof. He Huang, Dr. Yongxian Hu

# E-mail: huanghe@zju.edu.cn; E-mail: 1313016@zju.edu.cn

**Methods**

**Patients and data collection**

Patients with pathologically confirmed CD19+ ALL were enrolled in chronological order in clinical trials concerning CD19 CAR-T cells (ChiCTR-ORN-16008948) and CD19/CD22 CAR-T cells (ChiCTR1800015575) in the First Affiliated Hospital of Zhejiang University after approval by the ethics committee of our hospital. All patients during July 1, 2015 to April 30, 2020 were selected in this retrospective series. Clinical data were extracted from the electronic medical record system. Besides, flow cytometry data of CAR-T cells were collected from our laboratory. The grading of cytokine release syndrome (CRS) was based on the Penn grading scale^9^ and the grading of neurotoxicity was based on the Common Terminology Criteria for Adverse Events 5.0 (CTCAE 5.0).

**CAR-T cell generation and therapy**

The protocol of CAR-T cell generation in our center was described previously^6^. Briefly, peripheral blood mononuclear cells were obtained from patients by leukapheresis for transducing with CARs using lentivirus. The expansion and subtype of CAR-T cells were detected by flow cytometry after 2 day of transduction and on the day of infusion. All patients received Fludarabine- (30 mg/m^2^ on days 4–2) and Cy- (750 mg/m2 on day 2) based lymphodepletion regimen before CAR T-cell infusion. The expansion of CAR-T cells in vivo was calculated by continuously detecting CAR-T ratio in peripheral blood. Response rate was assessed using morphological analysis and flow cytometry analysis including CD10, CD19, CD20, CD34, CD38, and CD45 in 1 month after CAR-T therapy. Then patients were followed-up in out-patient departments for MRD detection in 2, 3, 6, 12, 18, 24, 36, and 48 months.

**Statistical analysis**

All measurement data were described using means with standard deviations and compared using t tests or described using median and range and analyzed using Mann–Whitney U test. Enumeration data were presented as frequency (%) and compared using chi-square tests or Fisher exact test. Odds ratio of risk factors associated with severe CRS was used logistic regression for univariate analysis. The analysis of overall survival (OS) used death as the event, the analysis of leukemia-free survival (LFS) used relapse as the event. Patients who did not have an event had their data censored for the last follow-up. Estimates and 95% confidence intervals (CI) for OS and LFS were calculated using the Kaplan-Meier method. All *P* values represented were two-sided and results were considered statistically significant when P < 0.05 (*). Data were analyzed using IBM SPSS Statistics 24. And Prism (Version 7.0) was used for drawing graphs.

**Figure Legends**

**Supplementary Figure 1.** a. The single chain fragment variable (scFv) sequence specific for CD19 was derived from Clone FMC63 murine and 4-1BB costimulatory domain and CD3ζ signaling domain were generated. And CD19/CD22 bispecific CARs were constructed by taking the standard four-domain CAR architecture. The antigen recognition domain of this CD22 specific CAR was obtained from a human antibody phage display library.

**Supplementary Figure 2.** a. Expression of scFvs of CD19 and CD19/CD22 CAR were detected by FACS after 2 days of transduction and the day of infusion. The box graph shows the minimum value to the maximum value and the lines from bottom to top present lower quartile, median, upper quartile value of transduction in individual group. b. Analysis of cell surface expression of CD45RA and CD62L on CAR-T cells, further gating CD4+ or CD8+ CAR-T cell on the day of infusion. The stacked bars describe means of different CAR-T subtypes.

**Supplementary Figure 3.** a-c. CAR-T cells in peripheral blood were continuously detected by FACS after infusion. The box graph shows the days that CAR-T achieved >1%, peak, <1% in peripheral blood. d. The peak number of CAR-T cell in peripheral blood was analyzed by complete blood count.

**Supplementary Figure 4.** a,c,d. Bars show the median with interquartile of minimum neutrophils, thrombocyte and hemoglobin. b. The box graph shows duration of neutropenia of two groups.

**Supplementary Figures and Tables**

**Supplementary Figure 1**


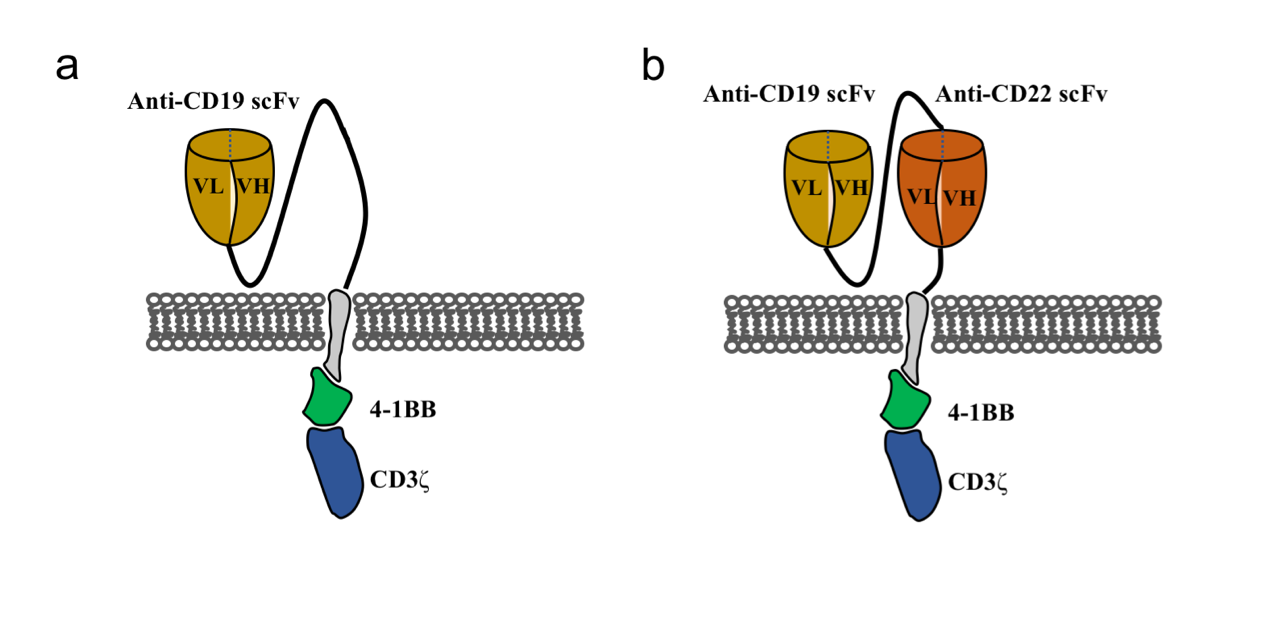


**Supplementary Figure 2**


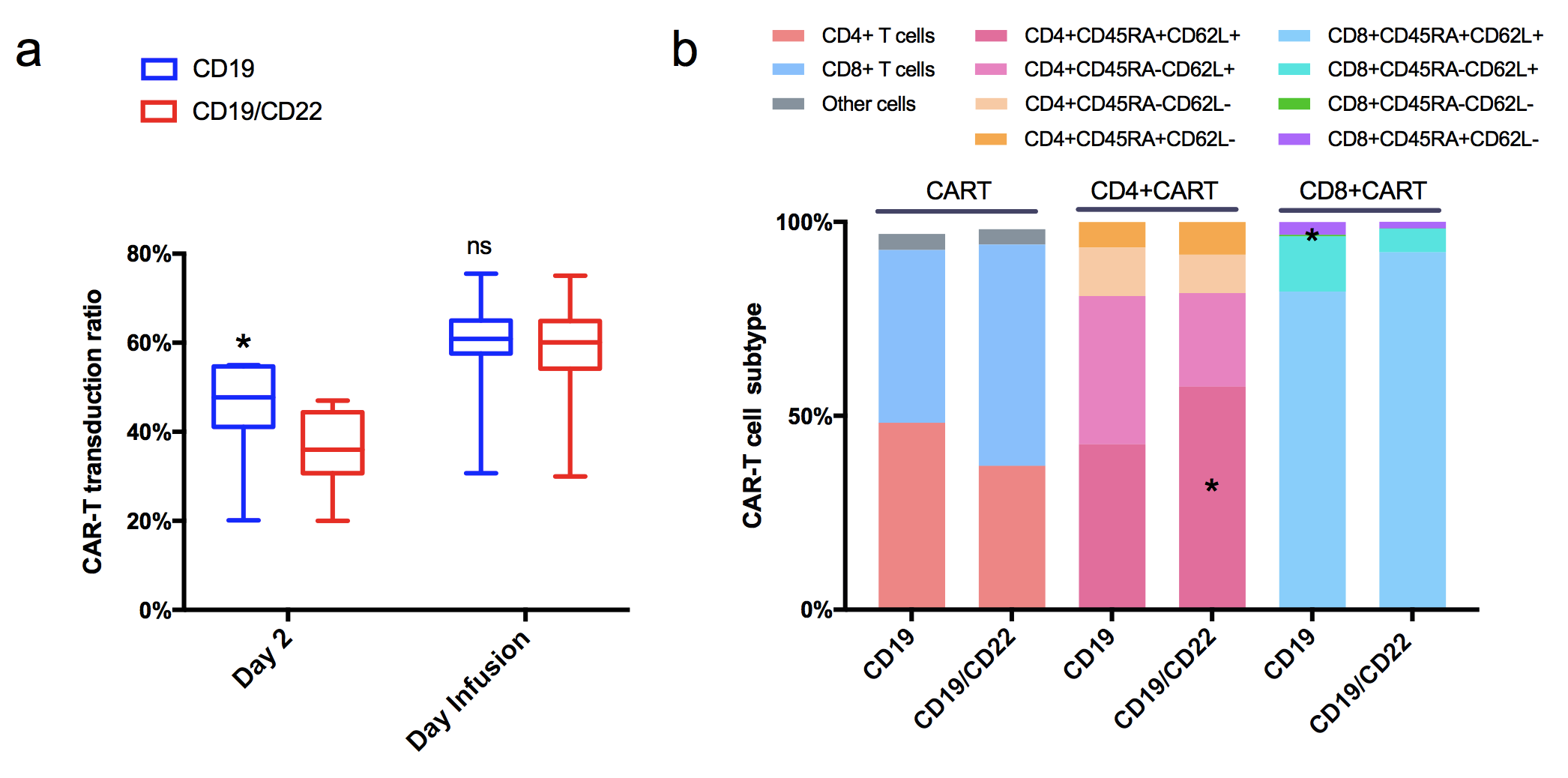


**Supplementary Figure 3**


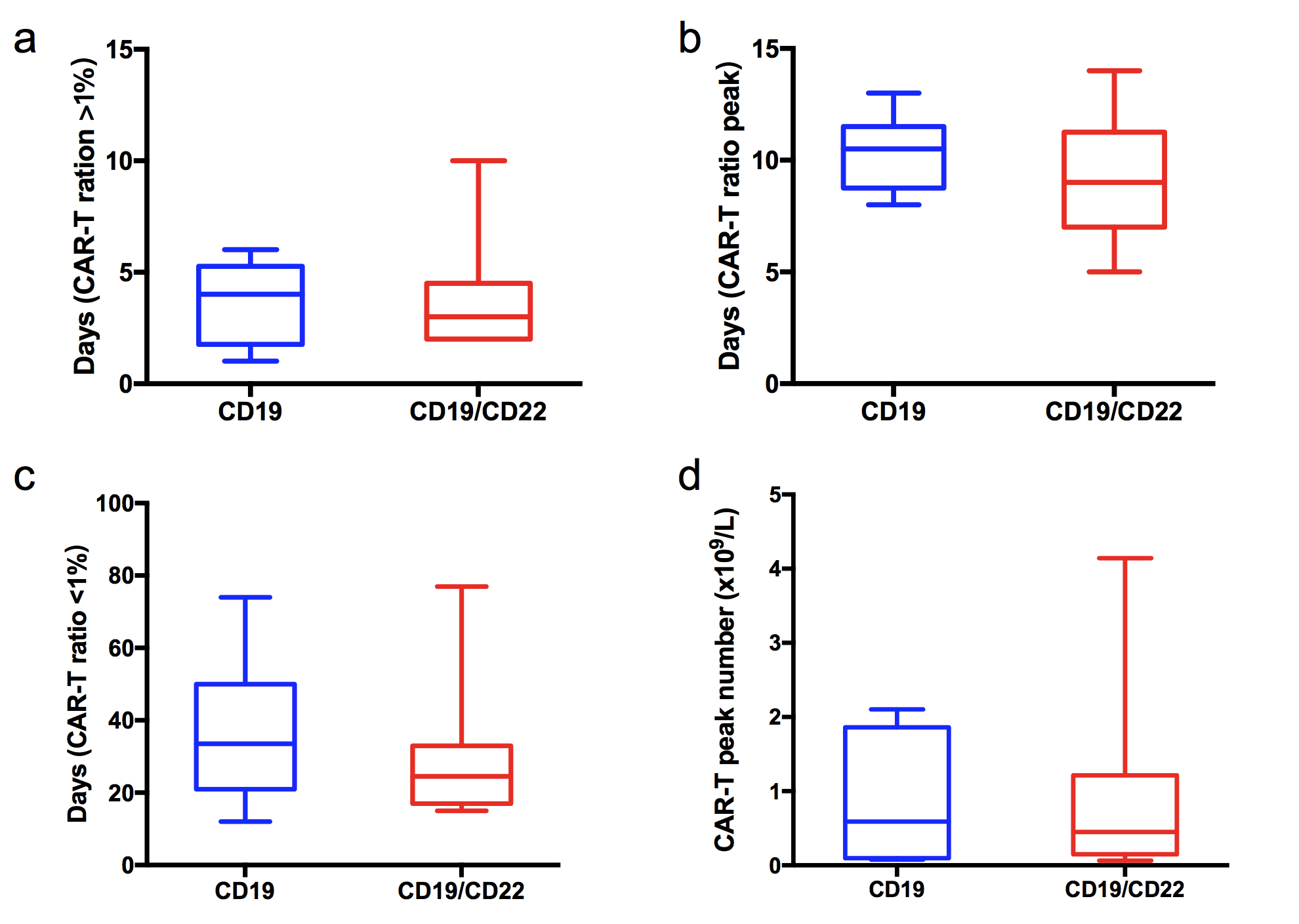


**Supplementary Figure 4**


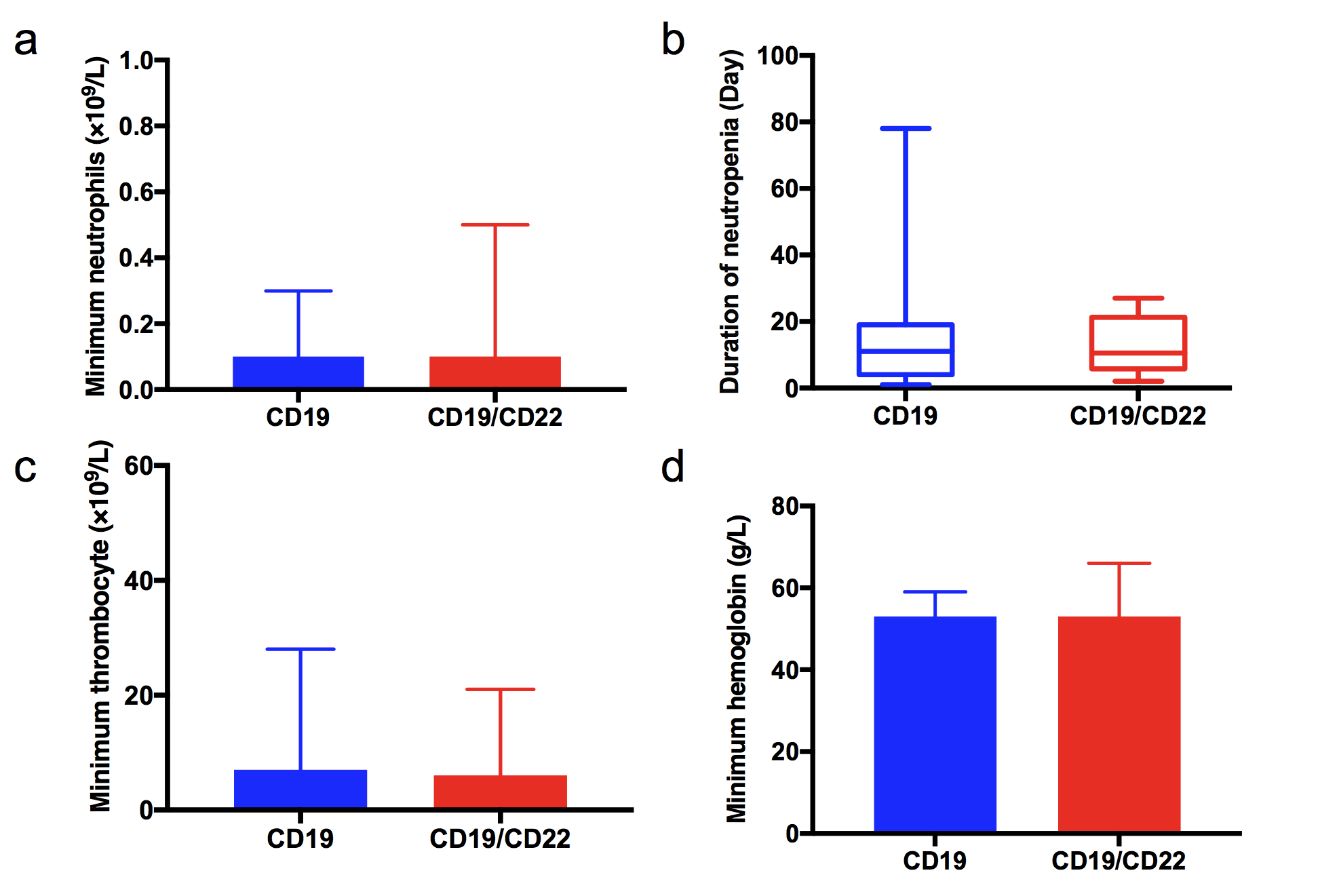


**Supplementary Table**

**Supplementary Table 1**

| Characteristic | CD19  (n=8) | CD19/CD22  (n=15) | *P* value |
| --- | --- | --- | --- |
| Gender, no. (%)  Female  Male | 5 (62.5)  3 (37.5) | 8 (53.3)  7 (46.7) | 1.000 |
| Age, range (year) | 29.5 (15-62) | 27 (16-65) | 0.797 |
| Hyperploidy, n (%)  Hypoplodiy, n (%) | 0 (0)  1 (12.5) | 0 (0)  1 (6.7) | /  1.000 |
| Complex karyotype, n (%)  BCR-ABL1, n (%)  ETV6-RUNX1, n (%)  E2A-PBX1, n (%) | 1 (12.5)  3 (37.5)  0 (0)  0 (0) | 1 (6.7)  4 (26.7)  0 (0)  1 (6.7) | 1.000  0.657  /  1.000 |
| KMT2A rearranged, n (%)  IKZF1 mutation, n (%) | 0 (0)  1 (12.5) | 1 (6.7)  1 (6.7) | 1.000  1.000 |
| Poor-risk cytogenetics, n (%)  Good-risk cytogenetics, n (%) | 4 (50.0)  0 (0) | 6 (40.0)  0 (0) | 0.685  / |
| Leukemia burden, range (%) | 33.5 (2-72) | 43 (4-84) | 0.423 |
| Prior numbers of therapy, range(no.) | 4 (1-6) | 5 (2-11) | 0.116 |
| Primary refractory to chemotherapy, n (%) | 1 (12.5) | 0 (0) | 0.348 |
| Numbers of relapses, range (no.) | 1 (1-2) | 1 (1-3) | 0.226 |
| Previous HSCT, no. (%)  Yes  No | 1 (12.5)  7 (87.5) | 1 (6.7)  14 (93.3) | 1.000 |

**Supplementary Table 2**

| Characteristic | CD19  (n=8) | CD19/CD22  (n=15) | *P* value |
| --- | --- | --- | --- |
| Transduction rate (%)  After 2 days  The day of infusion | 45.5±11.7  60.9 (30.7-75.5) | 35.9±8.9  60.1 (30-75.1) | **0.038**  0.438 |
| CD4+/CD8+ T-cell, range | 0.92 (0.57-2.70) | 0.52 (0.07-3.45) | 0.224 |
| Phenotype, range (%)  CD4+ Tnaive  CD4+ Tcm  CD4+ Teff  CD8+ Tnaive  CD8+ Tcm  CD8+ Teff | 30.6±29.0  38.2±19.0  12.5±9.2  92.9 (26.4-96.8)  2.75 (0.12-73.1)  0.24 (0.02-0.96) | 57.5±20.9  24.1±13.6  9.9±9.7  92.6 (77.8-98.8)  4.05 (0-22.1)  0.03 (0-0.28) | **0.024**  0.089  0.603  0.677  0.733  **0.029** |
| Expansion trend, range (day)  CAR-T cells >1%  CAR-T cells peak  CAR-T cells <1% | 4 (1-6)  10.5 (8-13)  33.5 (12-74) | 3 (2-10)  9 (5-14)  24.5 (15-77) | 0.548  0.444  0.353 |
| Peak CAR-T cell number,  range (×10^9^/μL) | 590.4 (76.0-2102.1) | 448.2 (63.5-4142.6) | 0.968 |

**Supplementary Table 3**

| Characteristic | CD19  (n=35) | CD19/CD22  (n=15) | *P* value |
| --- | --- | --- | --- |
| Gender, no. (%)  Female  Male | 21 (60.0)  14 (40.0) | 8 (53.3)  7 (46.7) | 0.662 |
| Age, range (year) | 31 (7-62) | 27 (16-65) | 0.896 |
| Hyperploidy, n (%)  Hypoplodiy, n (%) | 1 (2.9)  1 (2.9) | 0 (0)  1 (6.7) | 1.000  0.514 |
| Complex karyotype, n (%)  BCR-ABL1, n (%)  ETV6-RUNX1, n (%)  E2A-PBX1, n (%) | 3 (8.6)  8 (22.9)  0 (0)  1 (2.9) | 1 (6.7)  4 (26.7)  0 (0)  1 (6.7) | 1.000  1.000  /  0.514 |
| KMT2A rearranged, n (%)  IKZF1 mutation, n (%) | 2 (5.7)  3 (8.6) | 1 (6.7)  1 (6.7) | 1.000  1.000 |
| Poor-risk cytogenetics, n (%)  Good-risk cytogenetics, n (%) | 13 (37.1)  1 (2.9) | 6 (40.0)  0 (0) | 1.000  1.000 |
| Leukemia burden, range (%)  LDH, range | 33 (0.05-82)  282 (141-2805) | 43 (4-84)  260 (106-1964) | 0.343  0.285 |
| Prior numbers of therapy, range(no.) | 5 (1-24) | 5 (2-11) | 0.956 |
| Primary refractory to chemotherapy, n (%) | 5 (14.3) | 0 (0) | 0.305 |
| Numbers of relapses, range (no.) | 1 (1-6) | 1 (1-3) | 0.922 |
| Previous HSCT, no. (%)  Yes  No | 12 (65.7)  23 (34.3) | 1 (6.7)  14 (93.3) | 0.076 |
| CAR-T cell dose  , range(x10^6^/kg) | 3.2 (1.1-12) | 4.85 (1.04-7.02) | 0.374 |
| Tocilizumab only, no. (%)  Glucocorticoid only, no. (%)  Tocilizumab and Glucocorticoid, no. (%) | 8 (22.9)  4 (11.4)  3 (8.6) | 2 (13.3)  0 (0)  2 (13.3) | 0.702  0.302  0.629 |

**Supplementary Table 4**

| Factors | Odds ratio (95% CI) | *P* value |
| --- | --- | --- |
| CD19 or CD19/CD22 CAR-T | 0.183 (0.036-0.933) | **0.041** |
| Leukemia burden | 1.005 (0.984-1.027) | 0.624 |
| LDH | 0.999 (0.998-1.001) | 0.391 |
| CAR-T cell dose | 1.027 (0.805-1.311) | 0.828 |

**Supplementary Table 5**

| Characteristic | CD19  (n=35) | CD19/CD22  (n=15) | *P* value |
| --- | --- | --- | --- |
| CR rate, no. (%) | 32 (91.4) | 13 (86.7) | 0.629 |
| CRS, no. (%)  0-2 grade CRS  3-4 grade CRS | 19 (54.3)  16 (45.7) | 13 (86.7)  2 (13.3) | **0.029** |
| CRES, no. (%) | 2 (5.7) | 0 (0) | 1.000 |
| Duration of fever, range (day) | 8 (2-45) | 7 (3-40) | 0.348 |
| CRP, range (mg/L) | 63.8 (1.5-261.49) | 118.69 (36.49-314.6) | **0.017** |
| D-Dimer, range (μg/L) | 9639 (355-90000) | 84699 (475-88000) | 0.214 |
| Ferritin, range (ng/ml) | 6452 (36.8-40000) | 11761 (606-40000) | 0.675 |
| Cytokine, range (pg/ml)  IL-2  IL-4  IL-6  IL-10  TNFα  IFN  IL-17A | 3.48 (0.1-358.48)  2.23 (0.1-268.32)  190 (5.1-45314.0)  97.3 (4.82-3127)  2.39 (0.1-12671.8)  91.7 (0.1-6943)  4.47 (0.1-37.51) | 5.98 (0.24-75.99)  1.9 (0.1-15.6)  837.5 (54.5-12603.0)  95.3 (18.2-2301.6)  2.25 (0.1-34.2)  403.0 (5.71-4732.0)  9.33 (0.53-46.6) | 0.092  0.949  0.193  0.315  0.882  0.257  0.066 |
| Minimum C.B.C, range (×10^9^/L)  Neutrophils  Thrombocyte | 0.1 (0-1.4)  7 (1-175) | 0.1 (0-1)  6 (1-163) | 0.590  0.710 |
| Minimum Hemoglobin  , range (g/L) | 53 (32-129) | 53 (32-84) | 0.567 |
| Duration of neutropenia  , range (day) | 11(1-78) | 10.5 (2-27) | 0.622 |
| CAR-T cell peak numbers  , range (cells /μL) | 101 (23-2102.1) | 448 (63-4142.6) | 0.074 |
| OS, range (day) | 12 (38.7) | 9 (69.2) | 0.277 |

**Supplementary Table 6**

| Characteristic  （CR patients） | CD19  (n=31) | CD19/CD22  (n=13) | *P* value |
| --- | --- | --- | --- |
| No HSCT, no. (%)  Relapse  LFS, range (day)  No relapse  LFS (day) (95%CI) | 19 (61.3)  14 (73.7)  60 (30-261)  5 (26.3)  65 (0-183.0) | 4 (30.8)  3 (75)  90 (60-110)  1 (25)  90 (41-139) | 0.064  1.000  0.591  1.000  0.883 |
| HSCT, no. (%) | 12 (38.7) | 9 (69.2) | 0.064 |
| OS (day) (95%CI) | 364 (254.6-473.4) | 652 (399.0-905.0) | 0.198 |
